# Supplementary material for: Synthesis and Biochemical Evaluation of 8H-Indeno[1,2-d]thiazole Derivatives as Novel SARS-CoV-2 3CL Protease Inhibitors
Source: Molecules. 2022 May 23;27(10):3359. doi: 10.3390/molecules27103359 (PMC9145245; doi:10.3390/molecules27103359)
Supplement: Supplementary file 1 [file molecules-27-03359-s001.zip › molecules-1711411-supplementary.pdf]

Supplementary Materials

Synthesis and Biochemical Evaluation of 8*H*-Indeno[1,2-*d*]thiazole Derivatives as Novel SARS-CoV-2 3CL Protease Inhibitors

Jing Wu [1,†], Bo Feng [2,3,†], Li-Xin Gao [1,3], Chun Zhang [1], Jia Li [2,3,4], Da-Jun Xiang [5,*], Yi Zang [3,*] and Wen-Long Wang [1,*]

[1] School of Life Sciences and Health Engineering, Jiangnan University, Wuxi 214122, China;

[2] School of Life Science and Biopharmaceutics, Shenyang Pharmaceutical University, Shenyang 110016, China

[3] State key Laboratory of Drug Research, Shanghai Institute of Materia Medica, Chinese Academy of Sciences, Shanghai, 201203, China;

[4] Zhongshan Institute for Drug Discovery, Shanghai Institute of Materia Medica, Chinese Academy of Sciences, Zhongshan Tsuihang New District, Guangdong 528400, China;

[5] Xishan People's Hospital of Wuxi City, Wuxi 214105, China.

* Correspondence: Xishan People's Hospital of Wuxi City, Wuxi, 214105, China (D.-J. Xiang). State key La-boratory of Drug Research, Shanghai Institute of Materia Medica, Chinese Academy of Sciences, Shanghai 201203, China (Y. Zang). School of Pharmaceutical Sciences, Jiangnan University, Wuxi 214122, China (W. Wang). E-mail addresses: xiangdjxshospital@yeah.net (D. Xiang), yzang@simm.ac.cn (Y. Zang), wenlong-wang@jiangnan.edu.cn (W.-L. Wang).

† These authors contributed equally to this work.

Tables of Contents

Spectral data for compound **4** 3

Spectral data for compound **7a** 4

Spectral data for compound **7b** 5

Spectral data for compound **7c** 6

Spectral data for compound **7d** 7

Spectral data for compound **7e** 8

Spectral data for compound **7f** 9

Spectral data for compound **7g** 10

Spectral data for compound **7h** 11

Spectral data for compound **7i** 12

Spectral data for compound **7j** 13

Spectral data for compound **7k** 14

Spectral data for compound **7l** 15

Spectral data for compound **10a** 16

Spectral data for compound **10b** 17

Figure S1. 18

Figure S2. 18

^{1}H NMR of compound **4**

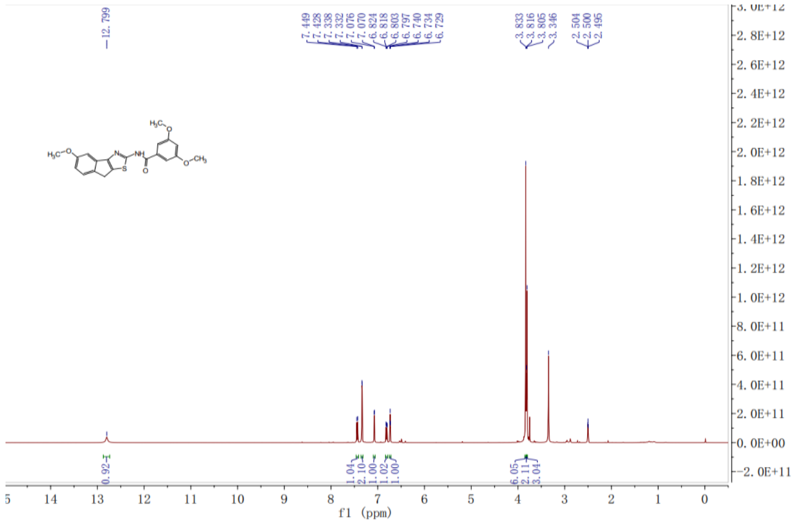

^{13}C NMR of compound **4**

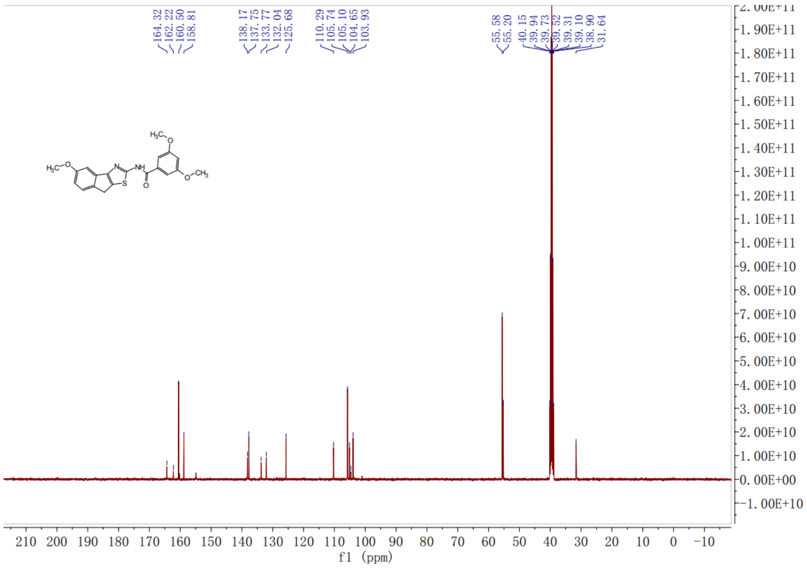

MS of compound **4**

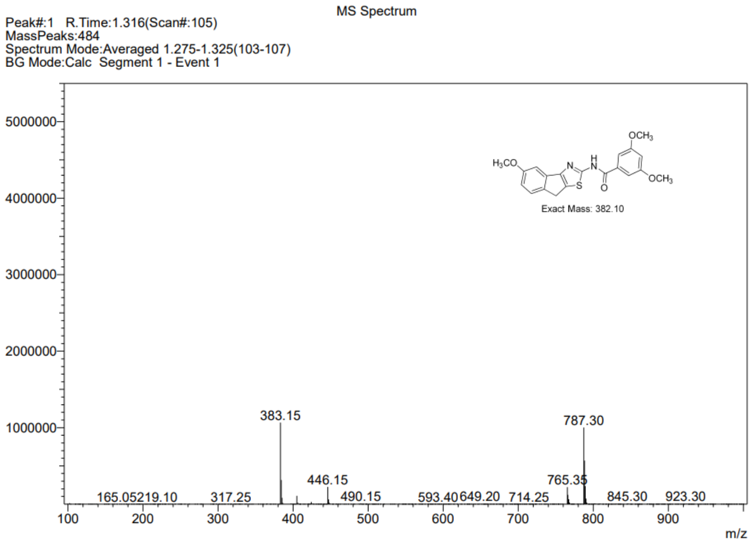

^{1}H NMR of compound **7a**

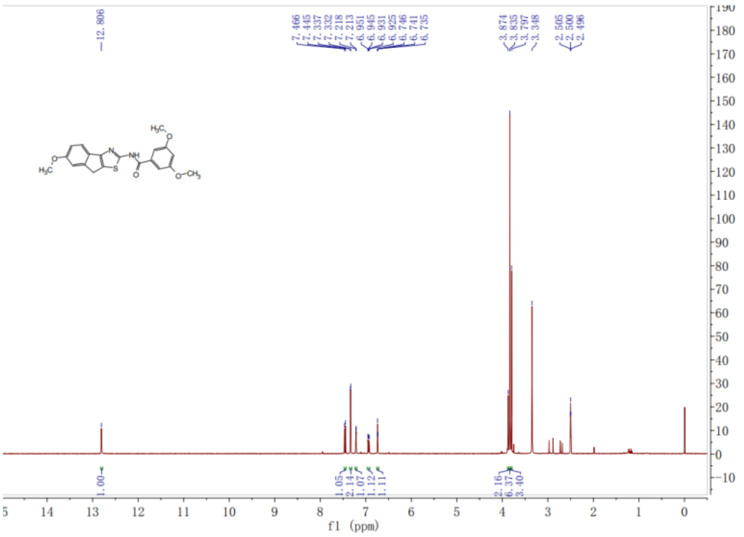

^{13}C NMR of compound **7a**

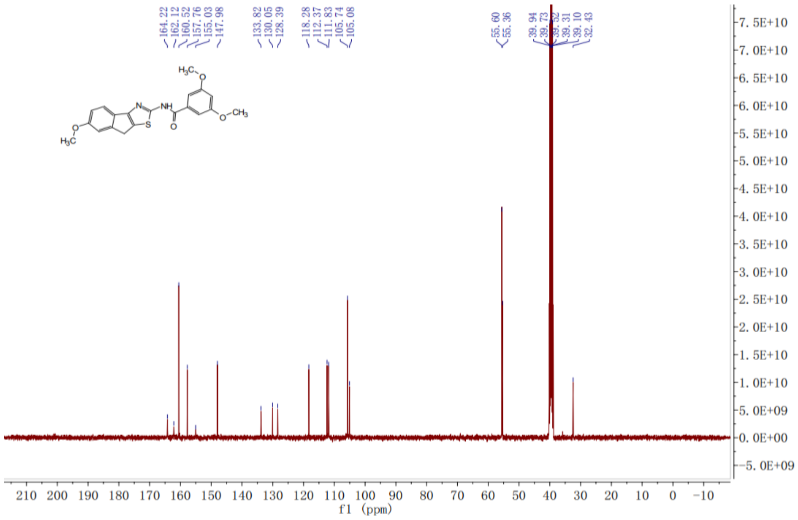

MS of compound **7a**

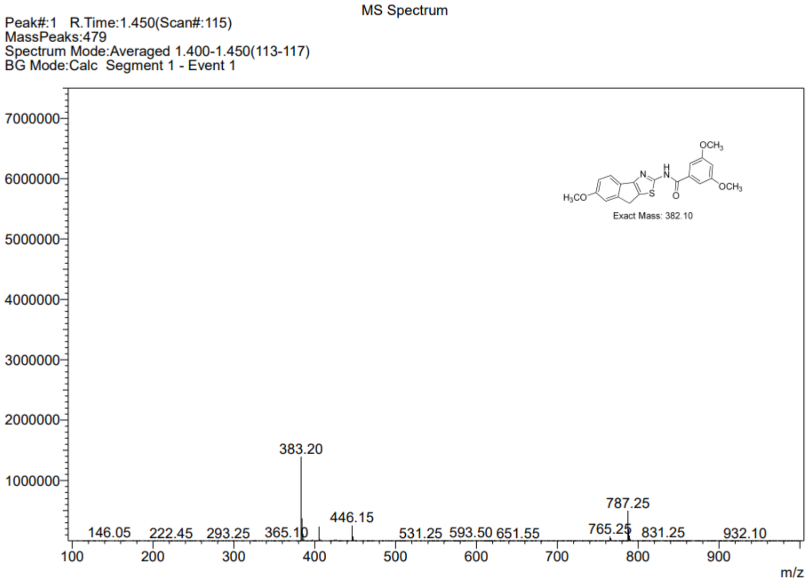

^{1}H NMR of compound **7b**

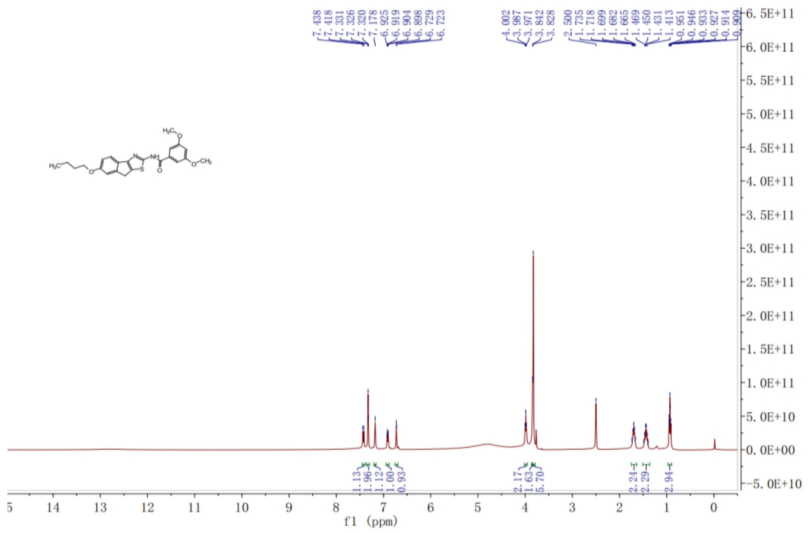

^{13}C NMR of compound **7b**

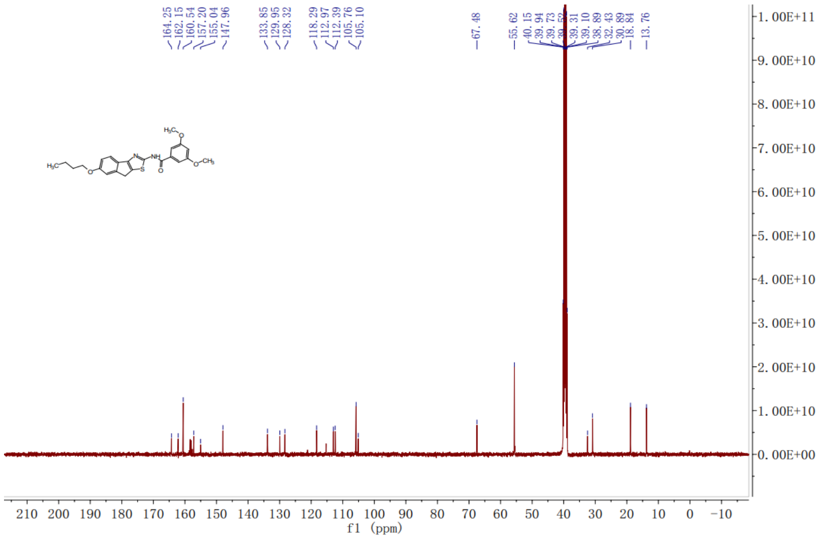

MS of compound **7b**

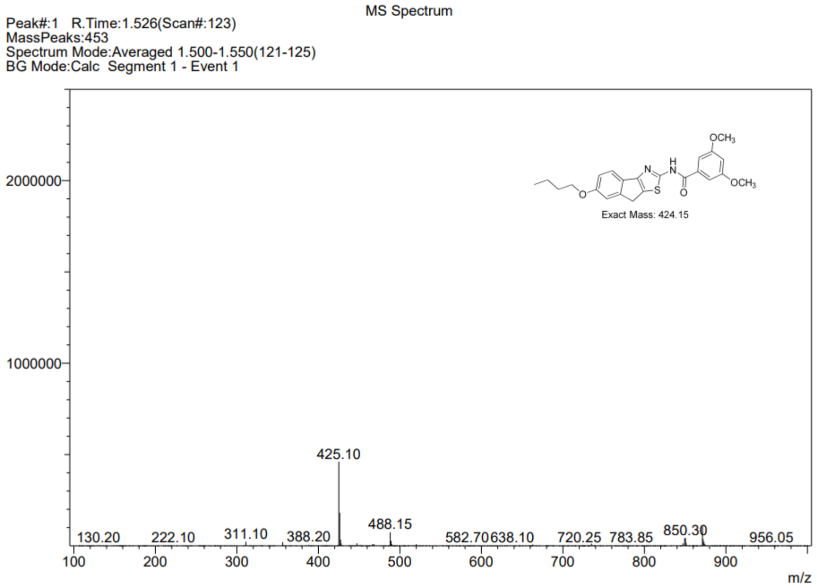

^{1}H NMR of compound **7c**

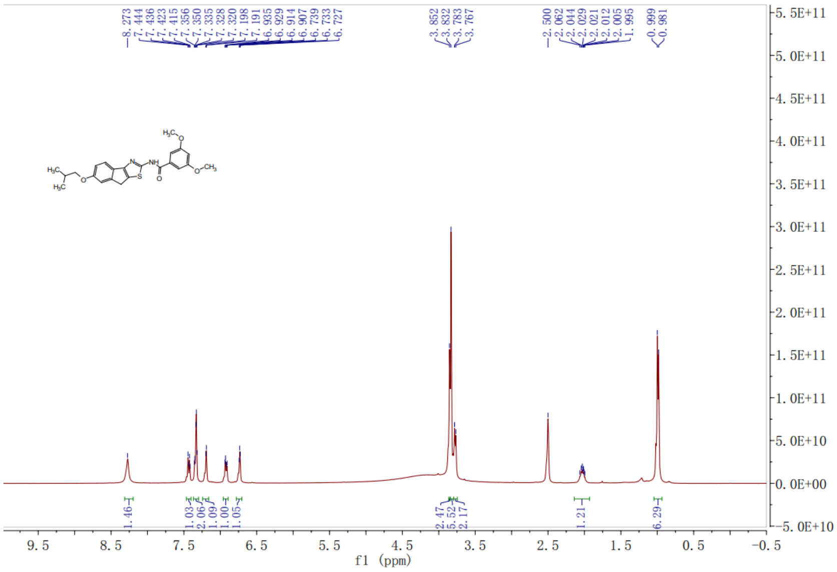

^{13}C NMR of compound **7c**

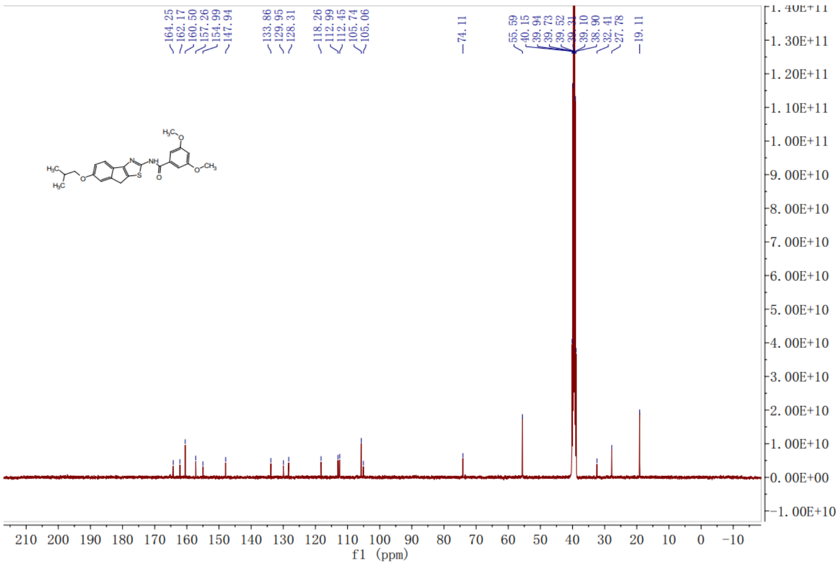

MS of compound **7c**

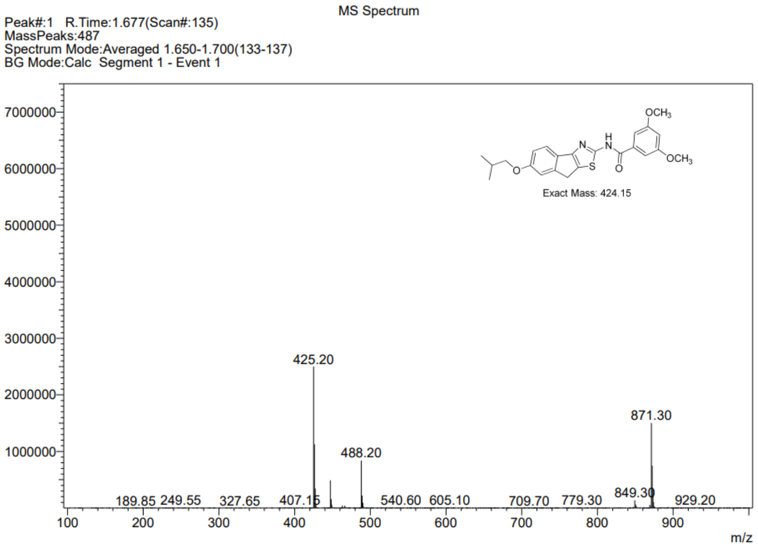

^{1}H NMR of compound **7d**

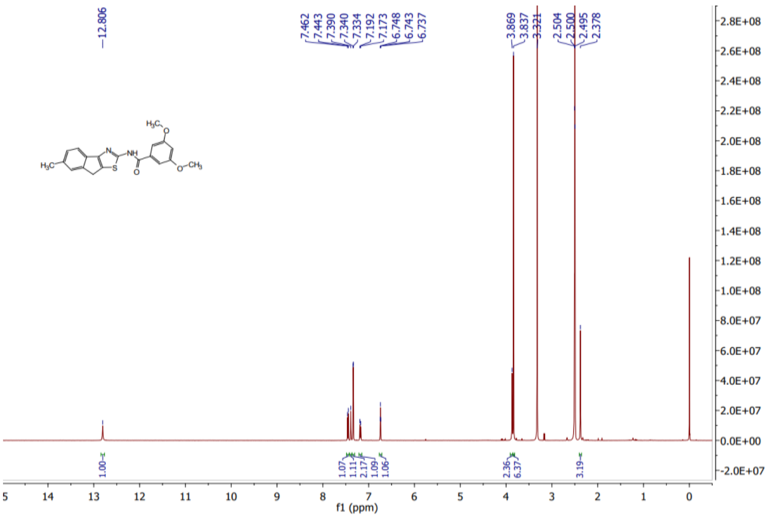

^{13}C NMR of compound **7d**

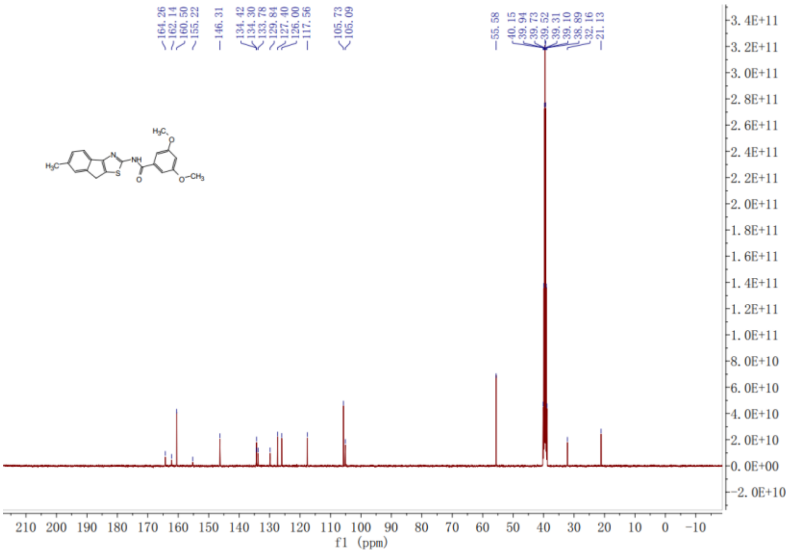

MS of compound **7d**

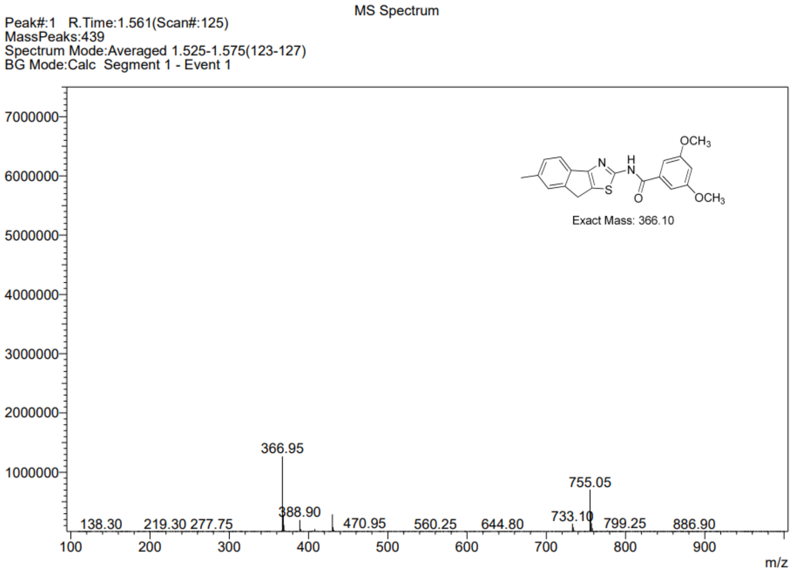

^{1}H NMR of compound **7e**

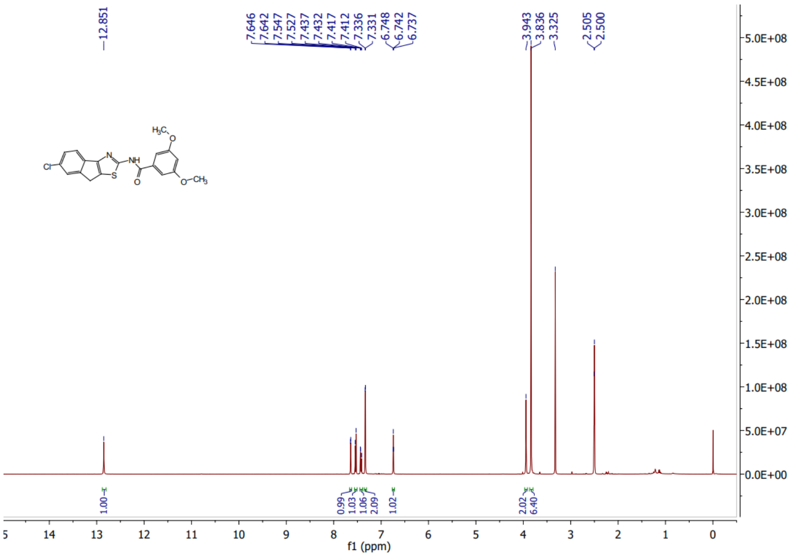

^{13}CNMR of compound **7e**

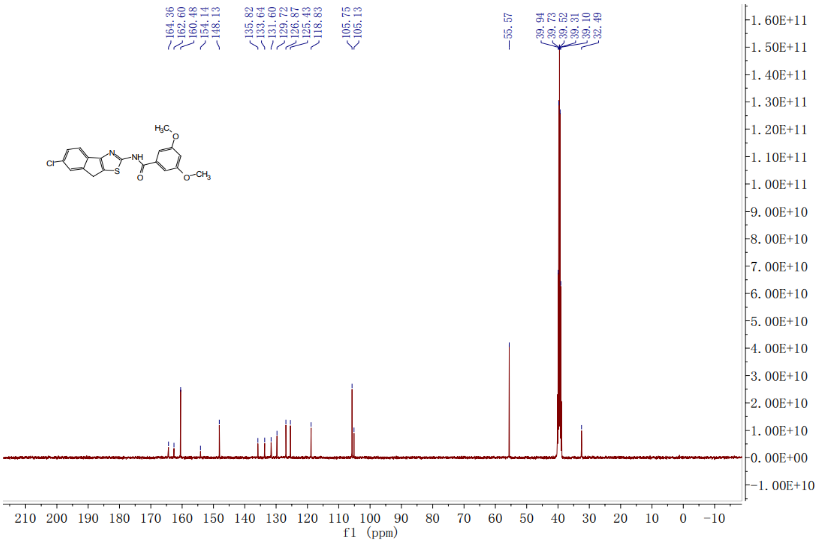

MS of compound **7e**

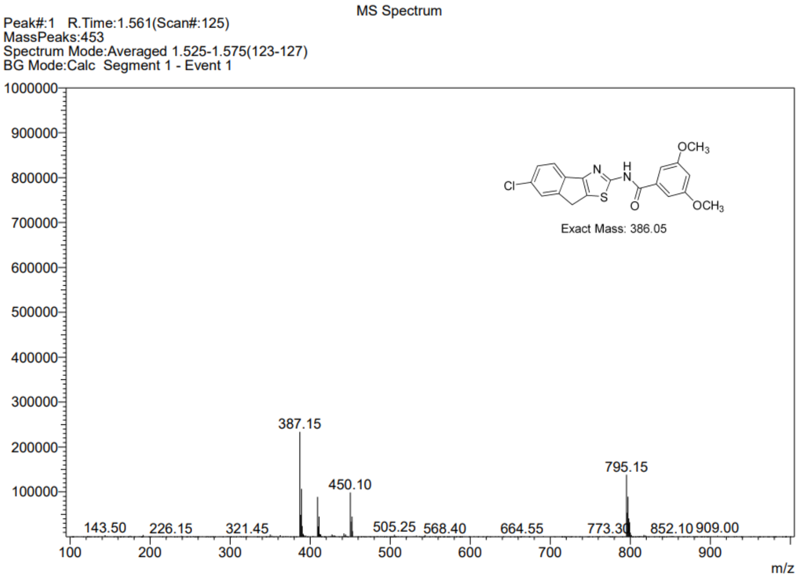

^{1}H NMR of compound **7f**

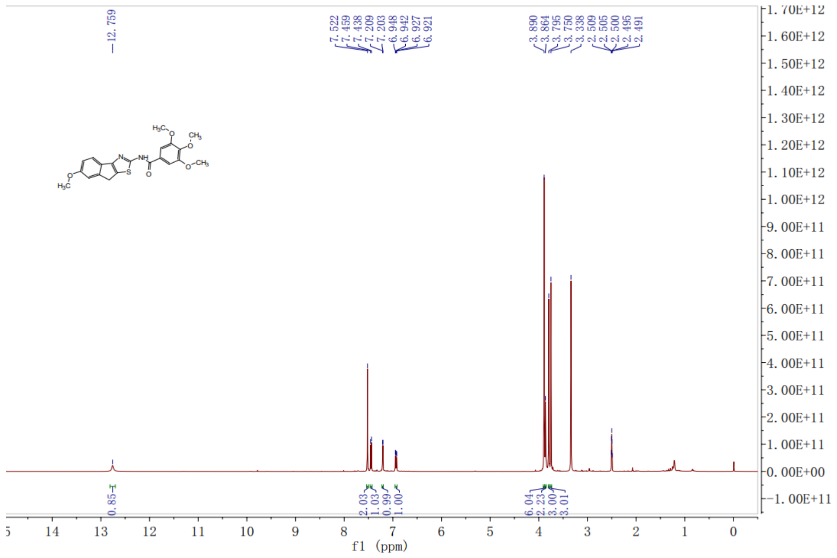

^{13}C NMR of compound **7f**

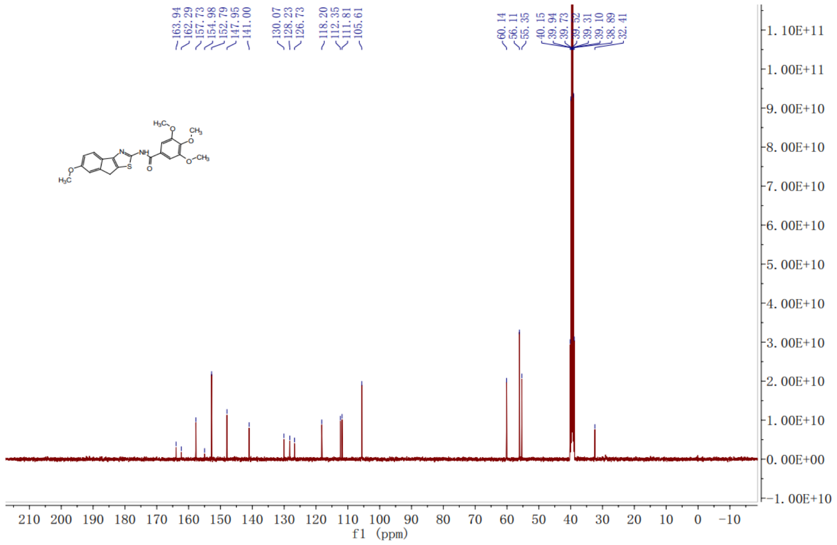

MS of compound **7f**

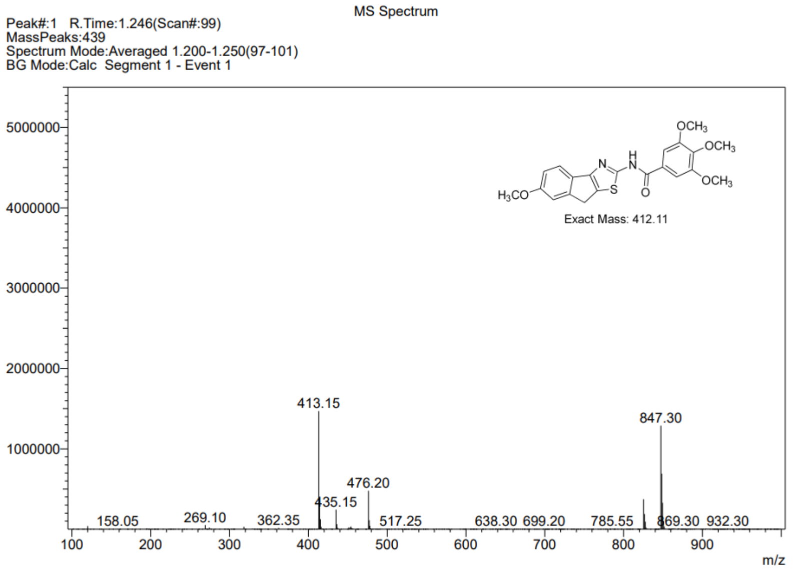

^{1}H NMR of compound **7g**

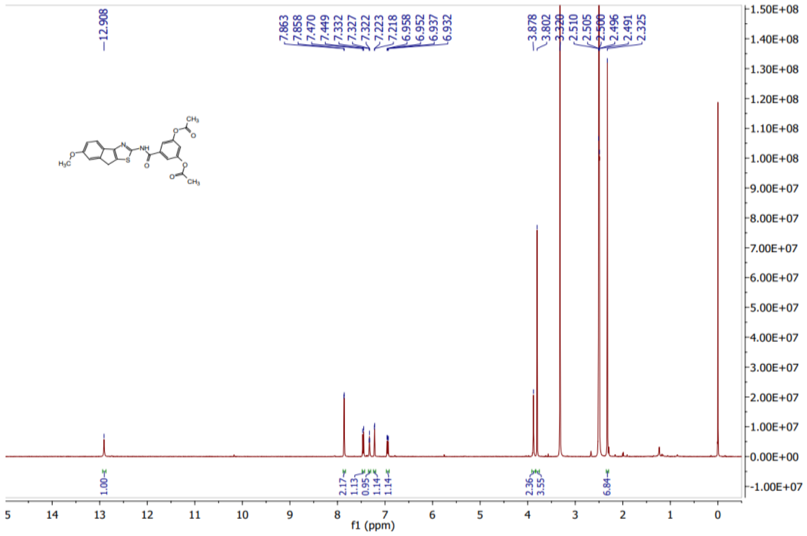

^{13}C NMR of compound **7g**

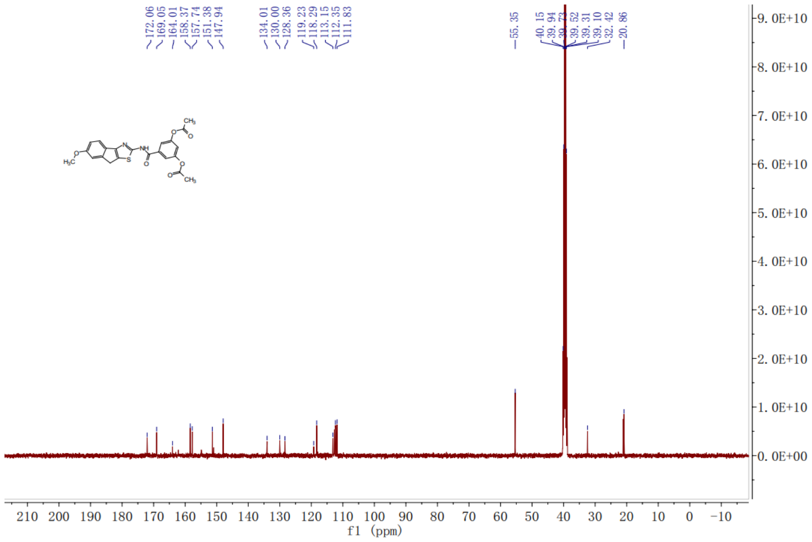

MS of compound **7g**

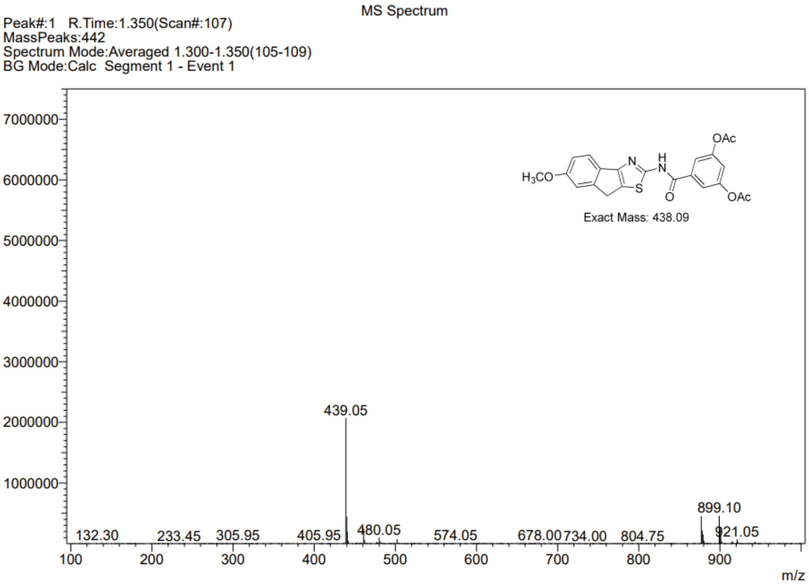

^{1}H NMR of compound **7h**

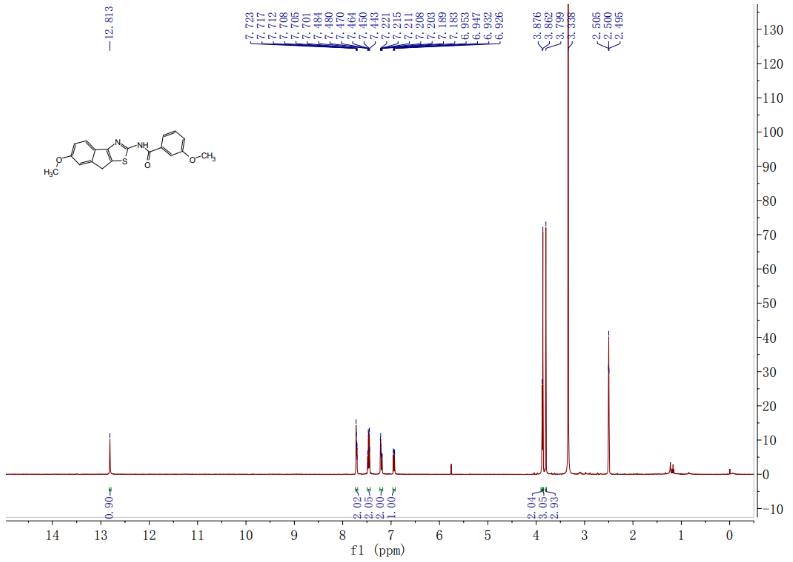

^{13}C NMR of compound **7h**

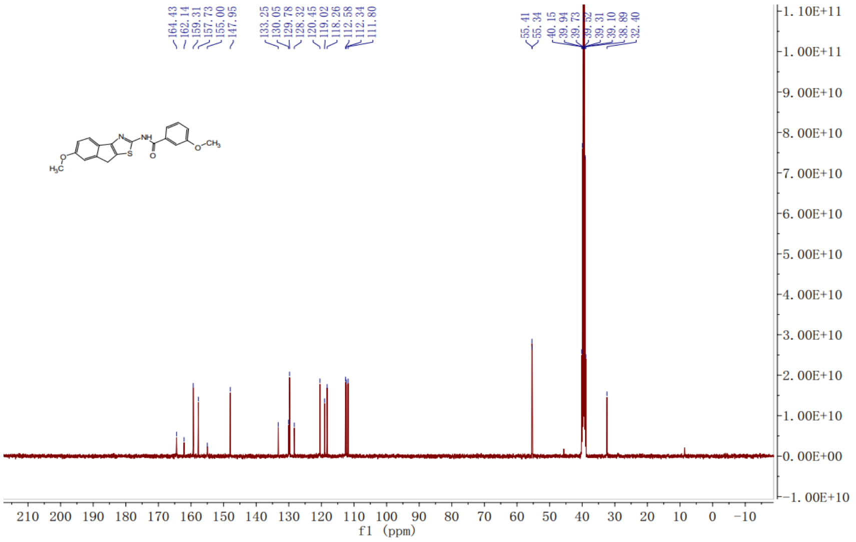

MS of compound **7h**

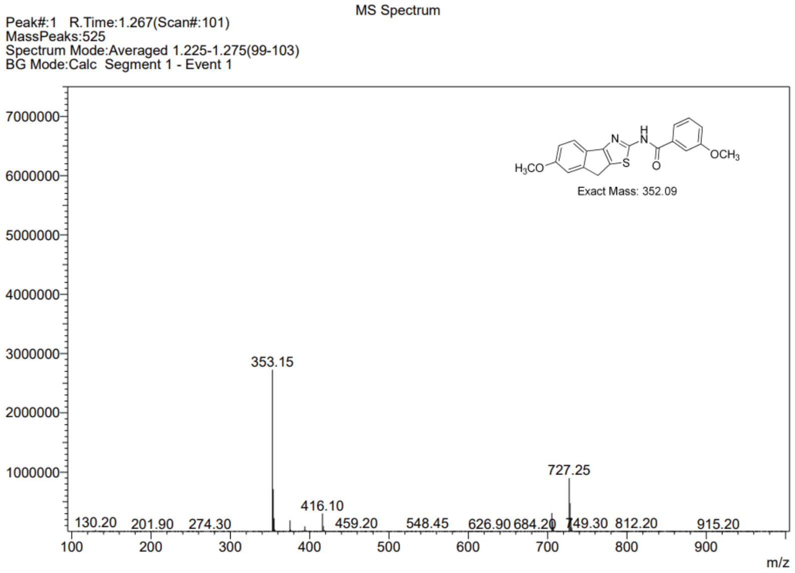

^{1}H NMR of compound **7i**

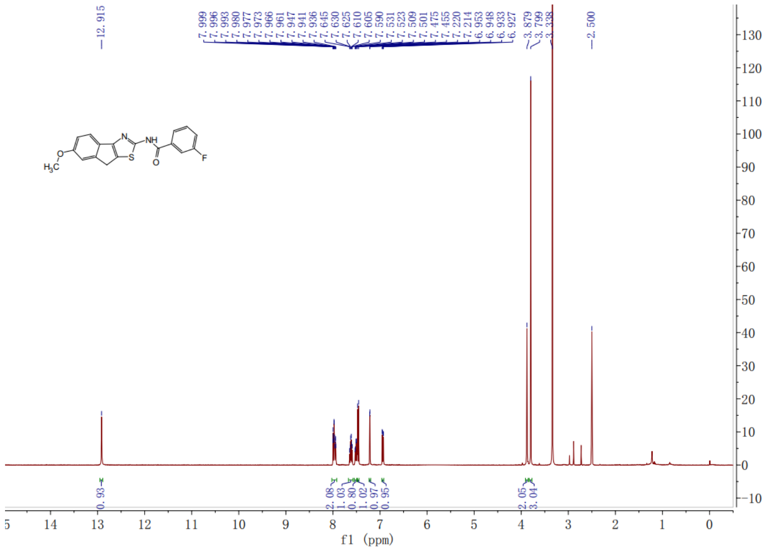

^{13}C NMR of compound **7i**

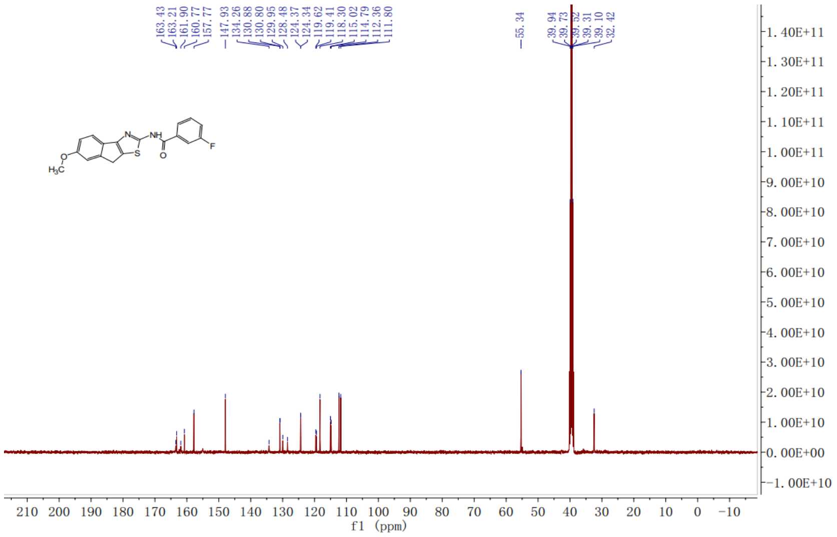

MS of compound **7i**

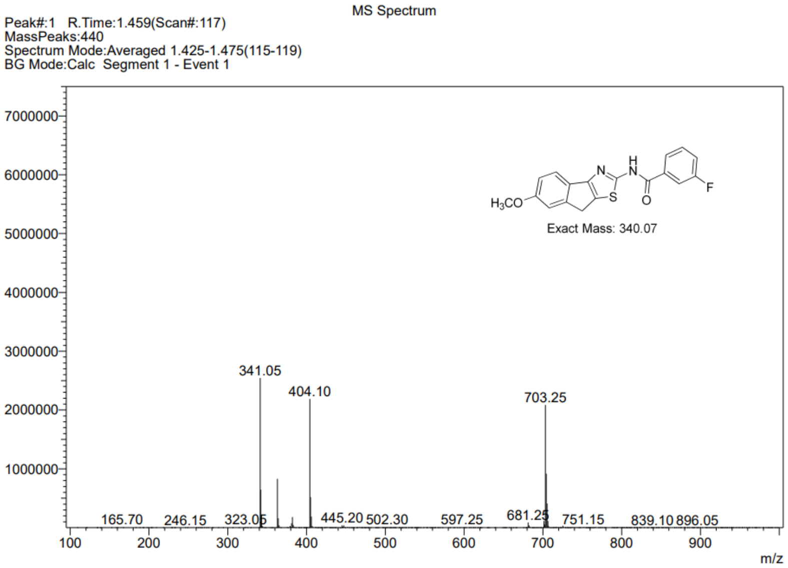

^{1}H NMR of compound **7j**

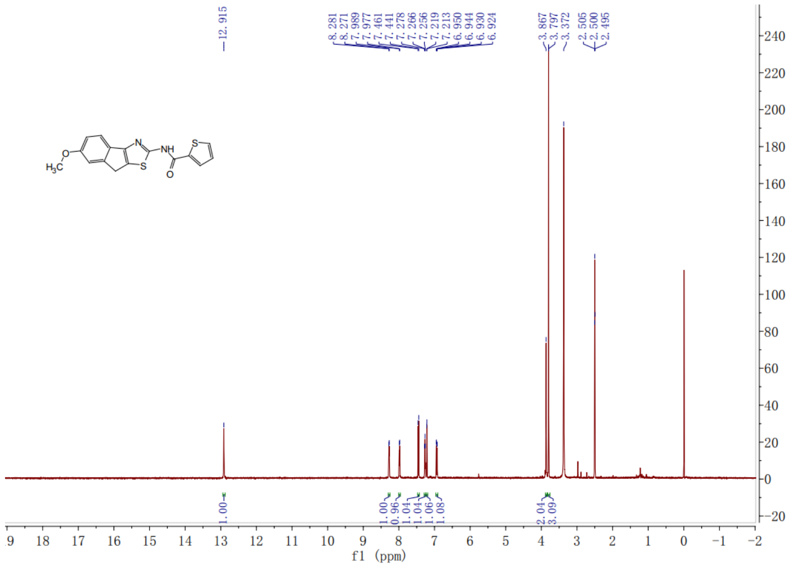

^{13}C NMR of compound **7j**

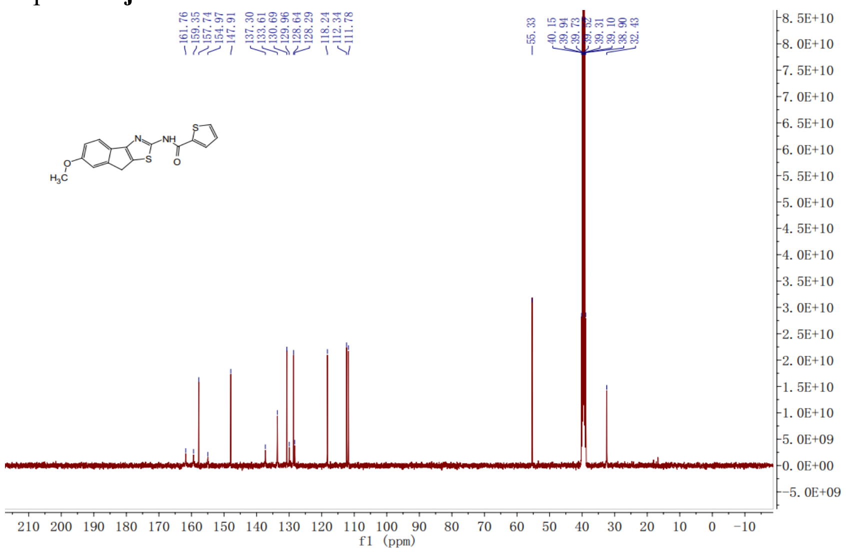

MS of compound **7j**

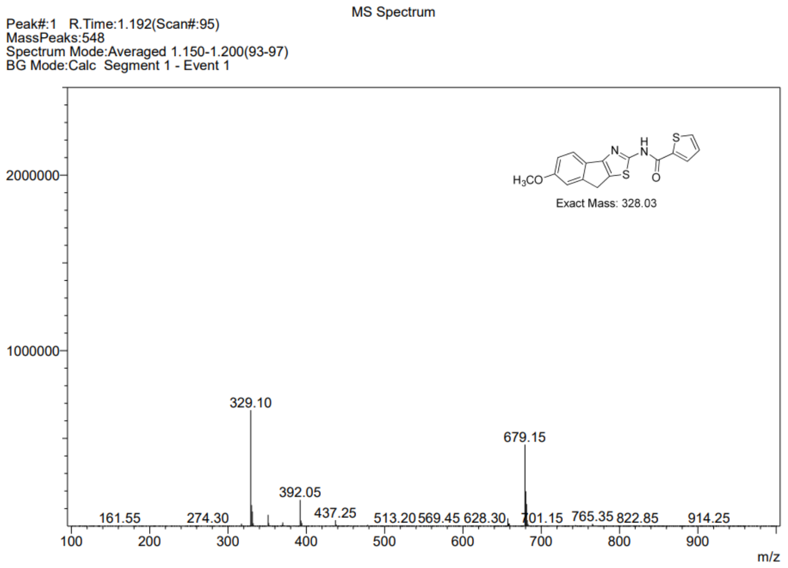

^{1}H NMR of compound **7k**

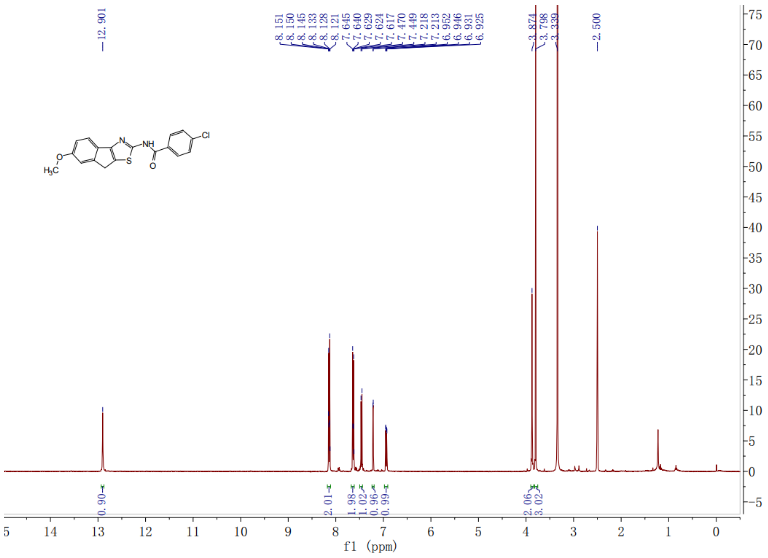

^{13}C NMR of compound **7k**

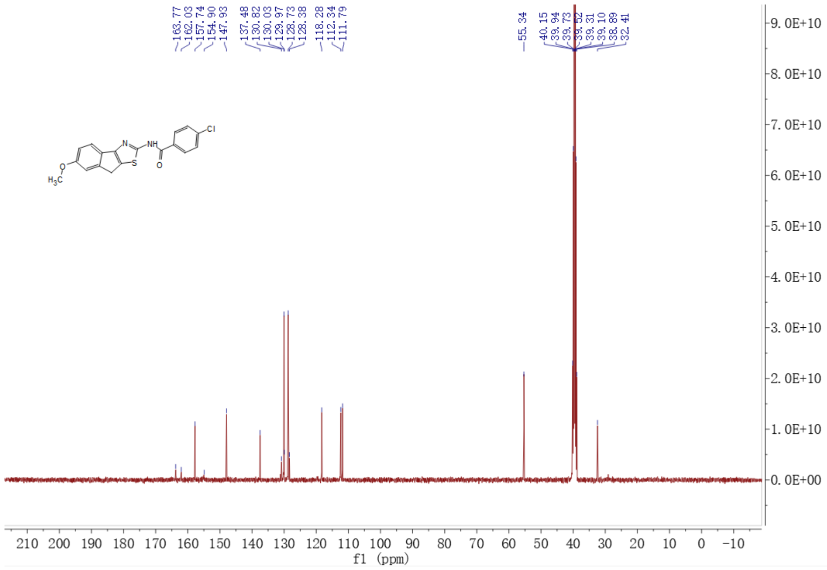

MS of compound **7k**

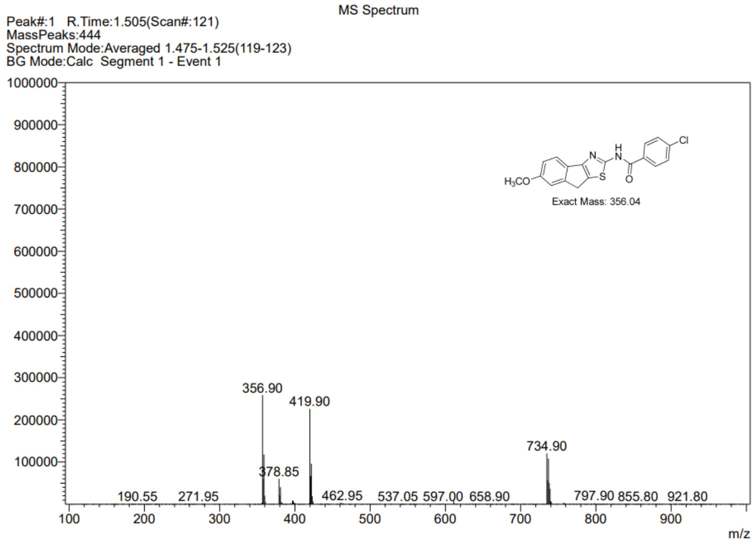

^{1}H NMR of compound **7l**

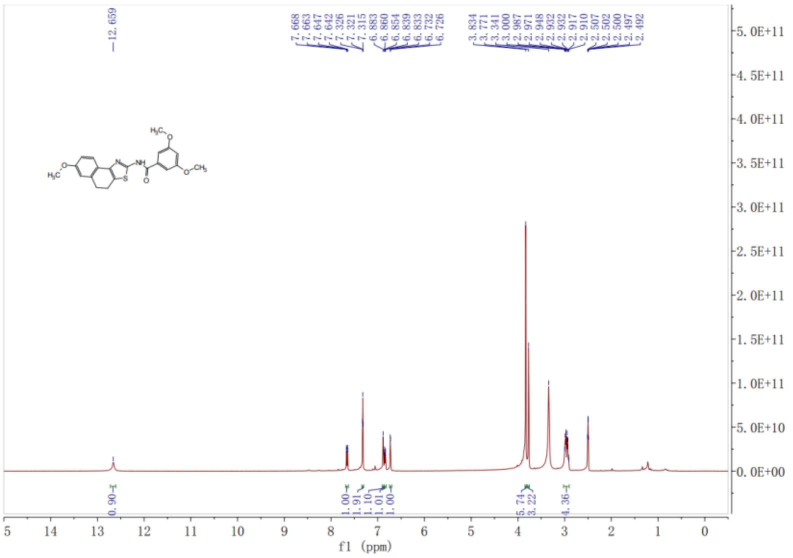

^{13}C NMR of compound **7l**

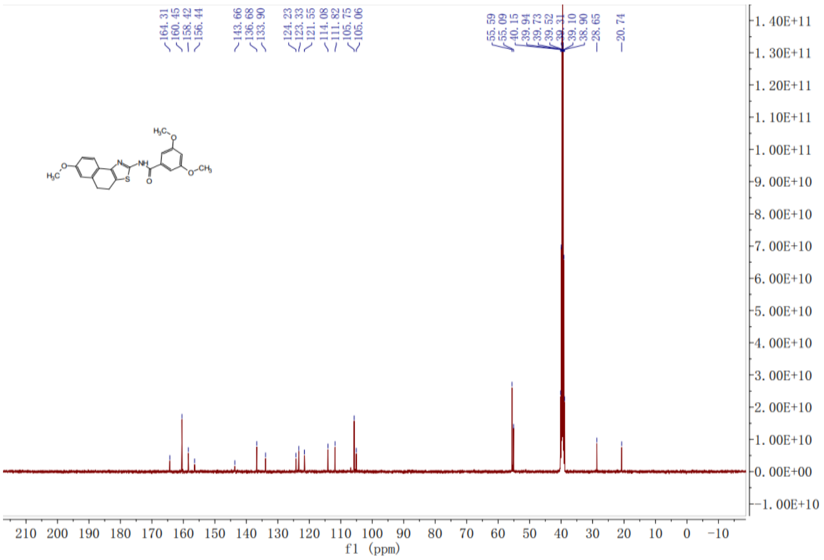

MS of compound **7l**

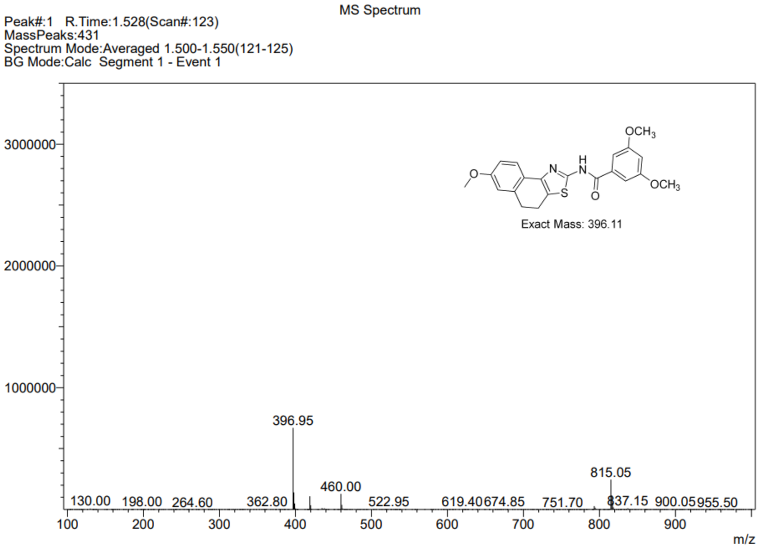

^{1}H NMR of compound **10a**

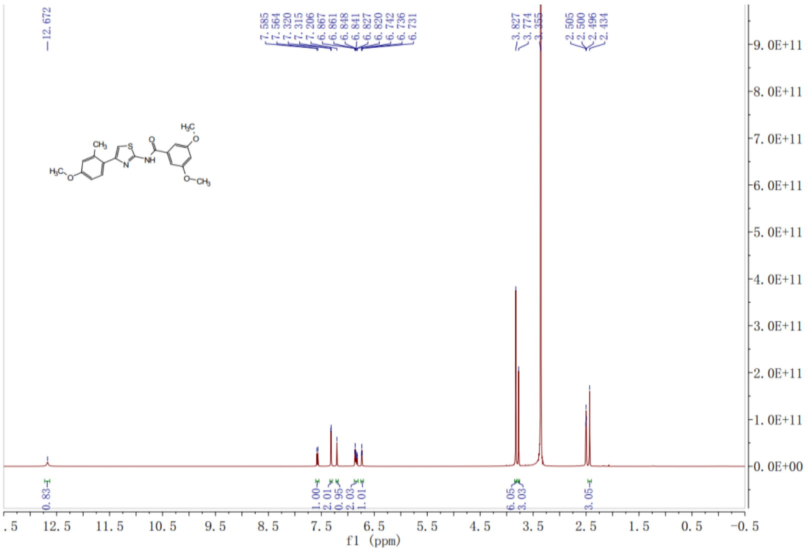

^{13}C NMR of compound **10a**

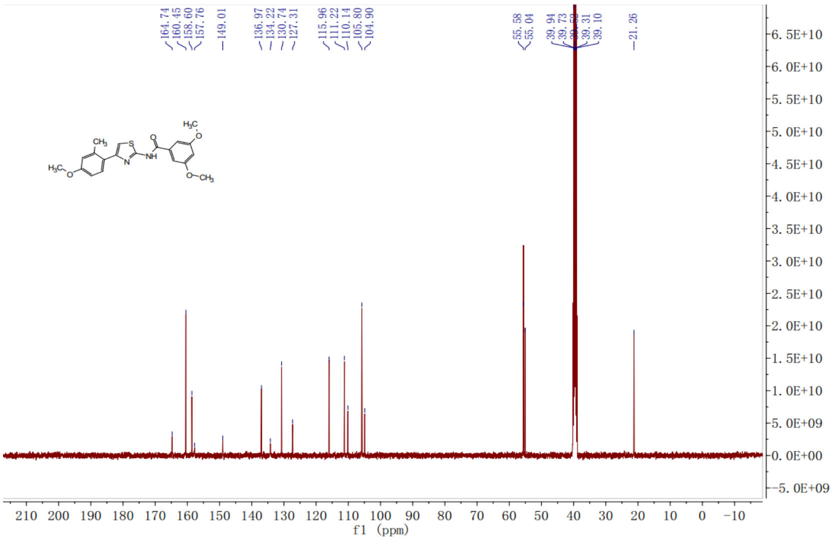

MS of compound **10a**

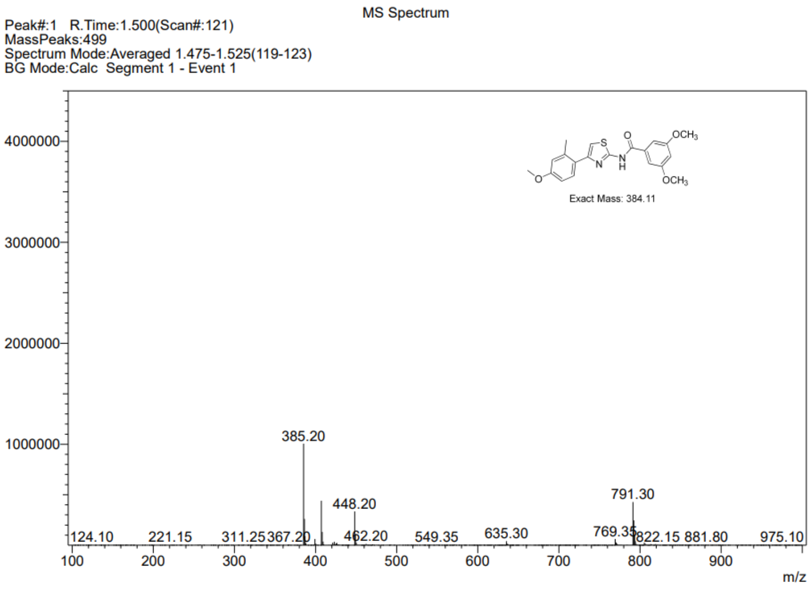

^{1}H NMR of compound **10b**

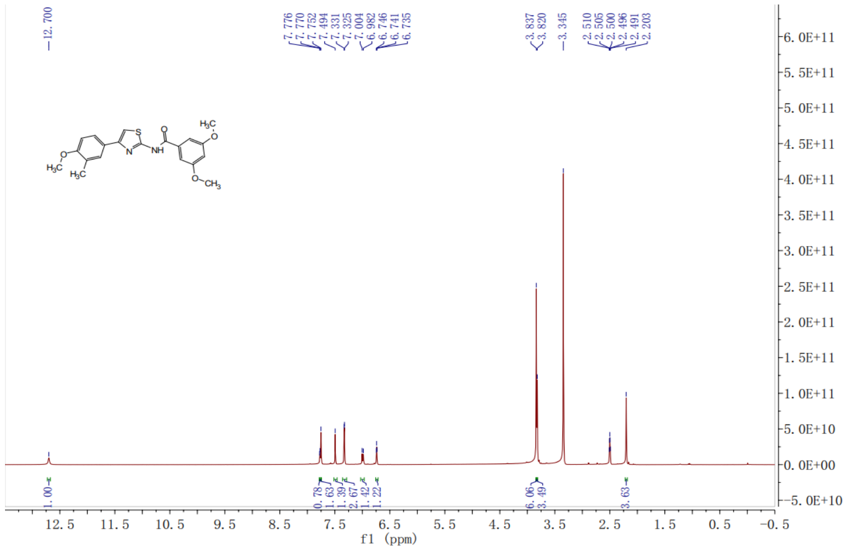

^{13}C NMR of compound **10b**

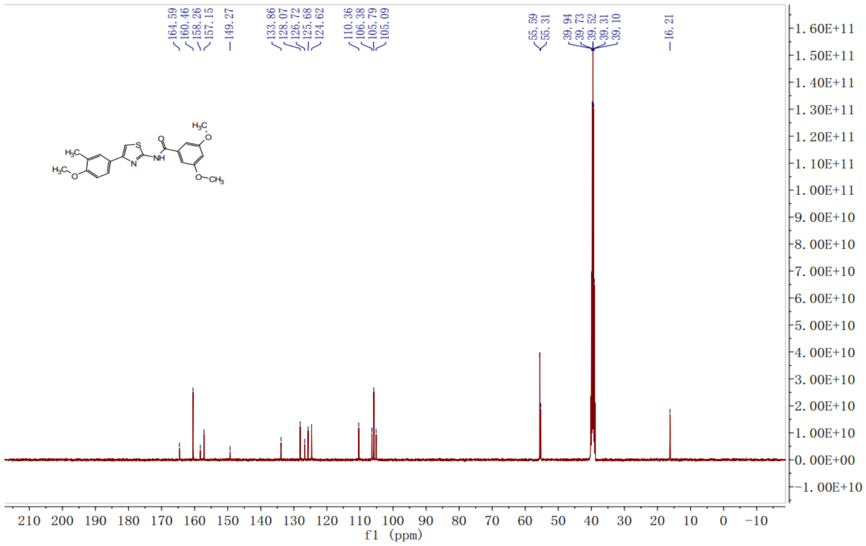

MS of compound **10b**

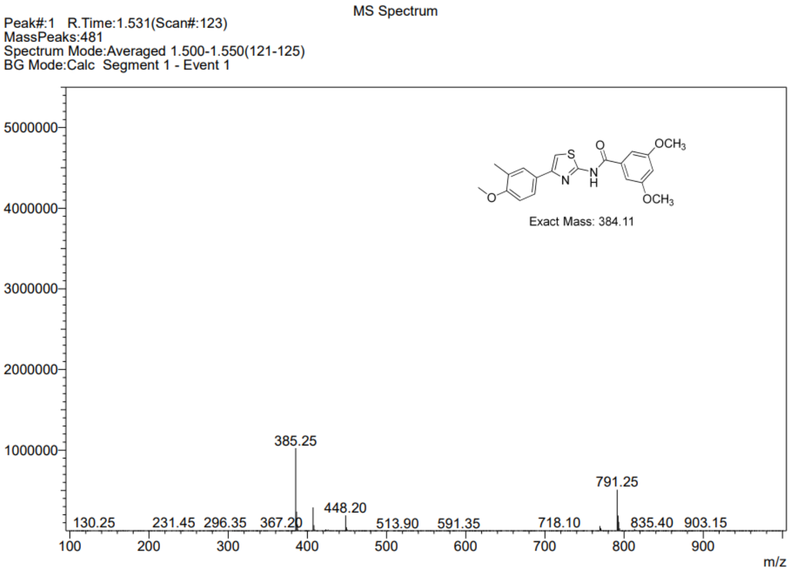

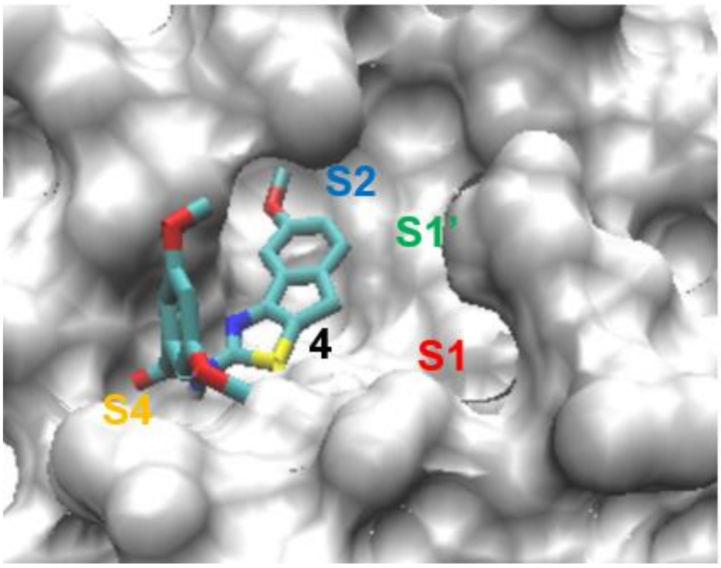

Figure S1. surf representation of the compound **4** (bonds representation) in the 3CL^{pro} S1(red), S2(blue), S1’(green), S4(orange) binding pocket

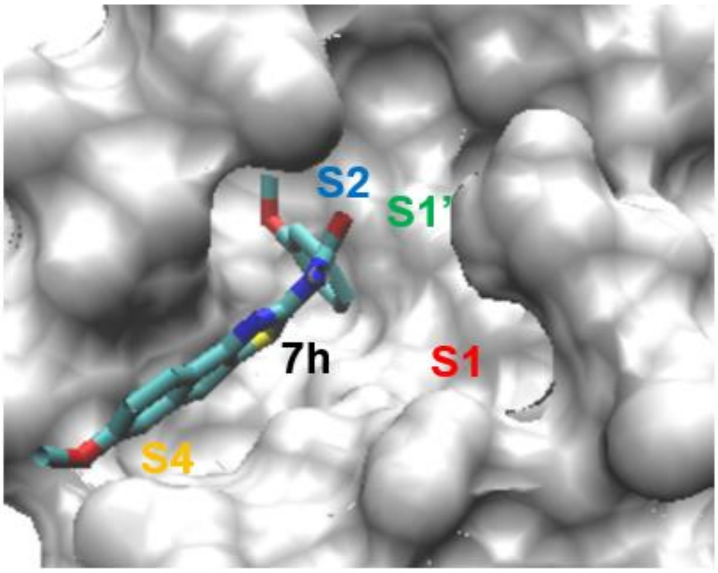

Figure S2. surf representation of the compound **7h** (bonds representation) in the 3CL^{pro} S1(red), S2(blue), S1’(green), S4(orange) binding pocket
